# Comment on “Metabolic Changes and Serum Ghrelin Level in Patients with Psoriasis”

**DOI:** 10.1155/2015/429429

**Published:** 2015-04-22

**Authors:** Mehmet Agilli, Fevzi Nuri Aydin, Tuncer Cayci, Yasemin Gulcan Kurt

**Affiliations:** ^1^Department of Biochemistry, Agri Military Hospital, 04100 Agri, Turkey; ^2^Department of Biochemistry, Sirnak Military Hospital, 73010 Sirnak, Turkey; ^3^Department of Medical Biochemistry, Gulhane Military Medical Academy, 06018 Ankara, Turkey

Thanks are due to Dr. Ucak and colleagues for their valuable research evaluating the levels of serum ghrelin in patients with psoriasis [1]. However, we wish to make some comments on methodology of their study.

Ghrelin is a hormone secreted mainly by stomach. Some types of antipsychotics, antidepressants, corticosteroids, bromocriptine, and anti-TNF-*α* therapy drugs were suggested to affect serum ghrelin levels [2, 3]. Also, dietary food supplements such as vitamin D and free fatty acids could affect these levels [4]. In this respect, the authors should define whether the participants use these kinds of drugs and dietary supplements or not.

In addition, several studies revealed that certain diseases such as functional dyspepsia, celiac disease, cachexia, inflammatory bowel disease, asthma, ankylosing spondylitis, coronary artery disease, iron deficiency anemia, hepatocellular cancer, chronic liver disease, chronic renal disease, epilepsy, and* Helicobacter pylori* infection could affect serum ghrelin [5, 6]. The authors did not mention these contributing diseases in their paper.

Ucak and colleagues stated that they questioned the patients' alcohol and cigarette smoking habits. As known, serum ghrelin levels are associated with alcohol intake and smoking [7, 8]. In this regard, a regression analysis could be applied for these variables to examine whether they affect serum ghrelin levels. Therefore, interpretation of results with its current form seems problematic.

In conclusion, we believe that the study of Ucak et al. contributes important data to medical literature and could lead other researchers to design a study. However clarifying above concerns will provide clearer picture to the readers.
